# Exploring stakeholder perceptions and priorities related to reducing tick-related public health risks in natural environments of the United Kingdom

**DOI:** 10.1186/s12889-025-24500-7

**Published:** 2025-10-02

**Authors:** Festus A. Asaaga, Emmanuel S. Tomude, Hermann Kam, Richard M. J. Hassall, Kayleigh M. Hansford, Saudamini Venkatesan, Maya Holding, Dominic P. Brass, Caroline Millins, Lucy Gilbert, Jolyon M. Medlock, Bethan V. Purse

**Affiliations:** 1 Present Address: UK Centre for Ecology & Hydrology, Maclean Building, Crowmarsh Gifford, Wallingford, OX10 8BB UK; 2 Medical Entomology & Zoonoses Ecology, UK Health Security Agency, Porton Down, UK; 3 Health Protection Research Unit in Environmental Change & Health, UK Health Security Agency, Porton Down, UK; 4 Institute of Infection, Veterinary and Ecological Sciences, University of Liverpool, Liverpool, UK; 5 Virology and Pathogenesis Group, UK Health Security Agency, Porton Down, UK; 6 Health Protection Research Unit in Emerging & Zoonotic Infections, UK Health Security Agency, Porton Down, UK; 7 Institute of Biodiversity, Animal Health and Comparative Medicine, University of Glasgow, Glasgow, UK

**Keywords:** Ticks, Tick-borne diseases, Land management, Co-production, Stakeholder attitudes, UK

## Abstract

**Background:**

Tick-borne disease (TBD) risks to humans and livestock are increasing rapidly in temperate regions, including the UK, with severe impacts on human and animal health and livelihoods. These threats could be exacerbated by large-scale policy-driven changes to increase woodland area and connectivity, which might increase the abundance of the key tick vector, *Ixodes ricinus*, and its interactions with people, livestock and wildlife hosts. Environmental interventions have been suggested as potential ecologically friendly options with the propensity to mitigate risk from ticks and TBDs in woodland areas, which are often characterised by high tick-human contact rates. Yet the uptake of these interventions is dependent on their alignment with stakeholders’ land management priorities and attitudes. Effective co-production practices will ensure that the implementation of land-based tick control interventions aligns with stakeholder priorities and are acceptable to society. However, there is limited empirical understanding about the viability and acceptability of land-based interventions to reduce risk from ticks on the ground.

**Methods:**

Three multi-stakeholder workshops (*N* = 40 participants), held in Aberdeenshire (Scotland) and New Forest (England) respectively, together with key-informant interviews (*N* = 18) were used to explore the stakeholders’ perceptions of, and attitudes towards, the potential implementation of land-based tick control interventions.

**Results:**

Overall, while study participants expressed a general concern about the tick ‘problem’, there were varied perspectives towards implementing potential environmental interventions for tick and deer management. Of the potential interventions identified, deer exclusion measures (e.g. fencing) were perceived negatively as costly and ineffective at scale. Study participants highlighted there is no one-size-fits-all intervention for deer and/or tick management, noting that a combination of interventions is needed to reconcile differing stakeholder priorities and expectations about land management on a landscape scale.

**Conclusion:**

Altogether, the study highlights the importance of harmonising land-use policies and continuous dialogue between stakeholders to reconcile competing land management objectives.

**Supplementary information:**

The online version contains supplementary material available at 10.1186/s12889-025-24500-7.

## Background

The spread of tick-borne diseases (TBDs) is on the rise globally, and increasingly seen as an emerging global public health concern [1, 2]. There is a clarion call for action as millions of people around the world, particularly those situated in close proximity to wooded habitats which are known to support high densities of tick vectors and key wildlife host of tick-borne disease pathogens are increasingly at risk of infection with endemic and emerging TBDs. This is especially so in temperate regions, including the United Kingdom (UK), where the recent spread and transmission of zoonotic TBDs (e.g. tick-borne encephalitis (TBE) and Lyme disease (LD)) have been linked to climate change and woodland expansion, increasing the exposure of humans to disease vectors’ habitats [3–5]. In the UK, for instance, reported cases of LD have increased 10-fold since 2000 [6], possibly linked to an expanding distribution of its main tick vector, *Ixodes ricinus* [4]. Tick-borne encephalitis virus (TBEV) is the causative agent of TBE, which can cause severe neurological disease and occasionally death with approximately 5,000–12,000 cases annually in Europe [7]. TBEV has a broad distribution and is established across much of mainland Europe and was first detected in the UK (England) in 2019 [8], followed by four probable or confirmed cases which were autochthonous [9].

While climate change mitigation and several government policies driving large-scale changes in landscape structure have merits for promoting ecosystem services (e.g. climate change adaptation and biodiversity conservation) and human well-being [10, 11], at the same time, there are potential public health ramifications with increasing amounts of habitats highly suitable for ticks, vectors and their hosts [3, 12–14]. Government policy in Scotland aims to increase woodland cover from 18 to 25% by 2050, requiring the creation of 10,000–15,000 hectares of woodland each year. In England, there is a corresponding target to increase woodland cover from 9 to 12% by 2060 [15]. Such changes in woodland cover and type are predicted to result in changes to host communities, tick populations and TBD risks to human and livestock health [12, 16, 17].

A wide spectrum of environmental interventions (such as deer exclusion and removal, vegetation control) have been suggested that have the propensity to reduce the risk of transmission of TBDs, particularly in woodland areas often characterised by high risk of tick-human contact [1, 5, 18, 19]. At the same time, societal acceptance of the implementation of environmental measures is observed to depend on the stakeholder priorities (e.g. increased woodland cover or biodiversity versus reduced deer populations), intended outcomes (e.g. reduced deer population or tick densities), the target species (e.g. deer, domestic livestock, birds), and the contextual conditions [4, 20, 21]. Differences in stakeholder attitudes towards the implementation of deer elimination (e.g. deer culling) and acaricide application at the landscape level have been observed [22]. For instance, some studies have reported that the implementation of deer management measures (e.g. deer culling) at scale is contested primarily due to ethical concerns around animal welfare standards [5, 23]. Nevertheless, there is some evidence that proper contextualisation (and consideration of associated trade-offs and win-wins and stakeholder priorities) could hold promise in optimising widespread uptake of evidenced-based environmental solutions across different socio-ecological, cultural, economic and political contexts [24, 25]. Since TBDs are a complex socio-ecological problem (in which multiple stakeholders with different interests and/or sectoral affiliations are implicated and affect the disease system) it necessitates a stakeholder-centered approach to better understand and reconcile conflicting objectives/priorities in contemporary land-use governance [1, 26, 27].

The global One Health paradigm, premised on the notion that health of humans, domestic animals and wildlife and the environment are linked and interconnected, provides a useful analytical entry point for evaluating these interactions in an integrated and cross-sectoral manner to reduce tick-borne pathogen transmission risk and mitigate disease impacts [28, 29]. However, critical scholarship has highlighted that current governance systems to manage ticks and TBDs spread is at best disjointed among key actors, with some local actor groups underrepresented and/or peripherally involved in landscape scale decision-making processes [26, 30]. One way of addressing such an issue is through stakeholder engagement in the co-production of knowledge for TBD management. This co-production process is underpinned by the argument that integration of different forms of knowledge about environmental interventions and/or stakeholder priorities is essential in the design and implementation of contextually acceptable interventions for reducing impact and probability of emergence of tick-borne zoonotic diseases [1, 31]. Proponents argue that this co-production process affords the conceptual space to engage knowledge holders in the research process as agents of transformative change where there is equitable knowledge exchange, fluid and permeable disciplinary boundaries [32, 33]. This approach is best suited for understanding of TBD dynamics and development of context-specific guidance and interventions, where stakeholders (e.g. policymakers, public health authorities, woodland owners and managers, foresters) from different sectors and scales are integral knowledge-holders and future users of risk guidance [26, 32, 34]. This is particularly compelling in the case of woodland management in the UK, where decision-making necessitates inclusive dialogue and negotiating trade-offs between different land management priorities with diverse stakeholders, who often do not normally act in concert [24, 32].

While extant studies have widely advocated the integration of diverse knowledge representations on risk factors to epidemiological models to inform TBD interventions [35], there is relatively limited exploration of stakeholder perceptions of ticks, TBDs and related environmental-based control options under different land management conditions, at least in the UK context [36]. Greig et al. [36], in their meta-analysis, for instance, found that of 2,258 articles on ticks and TBDs in North America, only 8.9% (202 articles) dealt with socio-behavioural drivers of TBDs. Understanding the spectrum of stakeholder interests and perspectives may strengthen land management decision-making around context-specific interventions that can account for unintended health consequences for diverse land use priorities and complex human-ecosystem interactions. We address the following research questions: (1) how do different woodland stakeholders and actors perceive risks from ticks and TBDs in relation to their land management priorities? (2) what do these perceptions imply for the implementation of potential environmental interventions to address TBD risks?

## Materials and methods

### Study contexts: new forest and Aberdeenshire

**Fig. 1:**
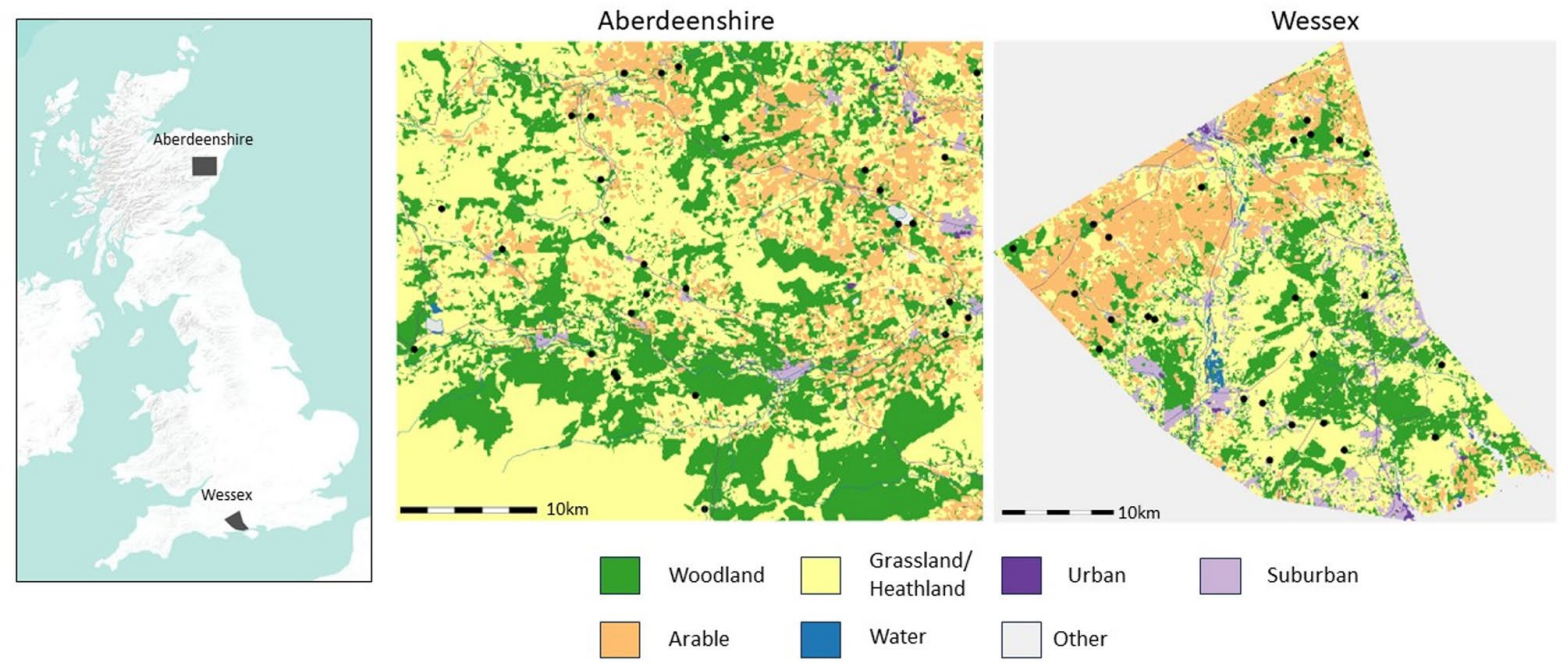
Map of study landscapes and sites with existing data on LD hazard (black dots) in the Aberdeenshire and New Forest regions. Map credit: TickSolve project [42], land classes depicted are from the UKCEH Land Cover Map [43], existing LD cases are unpublished data from UKHSA and University of Glasgow

Two mixed woodland landscapes, New Forest (southwest England) and Aberdeenshire (northeast Scotland) were purposively selected capturing a gradient of woodland patch sizes and connectivity, including potential environmental barriers and human usage as well as different histories of tick-borne diseases in people (Fig. 1). Attaining a national park status in 2005, the New Forest is a heterogenous landscape comprising unenclosed forest, heathland and grazed grassland with features of commoners grazing rights [37]. The region covers over 57,100 hectares and is a hotspot for human LD cases [38], possibly a reflection of human recreational use, high deer populations and infection prevalence in ticks [39]. In recent time, New Forest woodlands are undergoing restoration and expansion and is also a key emerging UK focus of TBEV [8]. Moreover, agricultural habitats between fragmented woodlands are subject to new environmental land management (ELM) schemes to increase habitat connectivity and biodiversity.

Aberdeenshire is also a LD hotspot with dense populations of primarily roe deer and high variance in Borrelia burgdorferi sensu (Bbsl) tick infection prevalence (up to 20%), dominated by small mammal-associated geno-species [40]. In keeping with the Scottish government policy, Aberdeenshire targets to increase woodland extent by 40% by 2050 to increase habitat networks and human access [11, 41].

### Stakeholder co-production process

We draw on multi-stakeholder (MS) framing workshops organised between 2022 and 2023 in the New Forest (southwest England) and Aberdeenshire (northeast Scotland) respectively to explore stakeholder perceptions and experiences about ticks and tick-borne disease management. These were conducted as part of a larger multidisciplinary research project on co-developing environmental interventions for mitigating spread and risks from tick-borne disease in UK woodlands [42]. Utilising a systems approach, we conceptualise the selected study regions as complex socio-ecological systems comprising human-modified ecosystems shaped by ecological, historical, political and economic processes. This approach affords a theoretical lens to capture all the dynamic interplay between different stakeholder groups whose activities and/or interests could impact significantly on land management in the study landscapes.

#### Recruitment of workshop and interview participants

Leveraging a detailed stakeholder mapping, analysis and assessment by the project team, workshop participants and key-informants were purposively selected to cover the key roles on the agenda setting and implementation of land management interventions at the landscape scale. These target stakeholder groups were selected based on their experience working on environmental and land management issues in the study landscapes, and stratified by sector (land, biodiversity and wildlife) to capture occupational heterogeneity [32]. These groups were considered to have the requisite contextual knowledge to share and have a direct “stake” and/or interest in land management and/or agricultural decision-making in the study contexts. An open request to participate was emailed to eligible groups whose work or leisure activities involve spending time in areas where ticks may be present, such as landowners, farmers, estate managers, and foresters leveraging the research team’s extensive networks. (see key-informant characteristics in Additional file 1).

#### Multi-stakeholder workshops

Three problem framing workshops were designed to elicit local stakeholders’ perceptions about ticks and tick-borne diseases and prioritise contextually feasible and appropriate land-based interventions for reducing TBD risks. We employed a participatory consensus building technique [32] in the MS workshops. In this context, the first workshop was held at Fordingbridge in the New Forest region in November 2022. The second workshop (which targeted stakeholders in Aberdeenshire, *n* = 6 participants) held online on 8th March 2023. The third workshop (which targeted local policy actors and woodland management groups, *n* = 20 participants) was held at the Lymington Town Hall on 3rd May 2023. The three workshops aimed to map stakeholders’ knowledge about ticks and TBDs and prioritisation of potential interventions for control with the view of feeding that knowledge into project approaches and models. To this end, each workshop had facilitated discussions and simple voting process (rank ordering risk factors and needs) of the nominal group technique (NGT) to help overcome the power dynamics of diverse land use groups and afford a more inclusive group discussion [32]. A thematic workshop guide was developed to help guide the discussions (see Additional file 2). Participants were pre-organised into groups (3 for New Forest-workshop 1, 4 for New Forest-workshop 2 and 2 for Aberdeenshire respectively) reflecting organisational affiliation/occupational backgrounds to allow for the cross-fertilisation of ideas and exchange. The two in-person workshop discussions (i.e. both held in the New Forest) were recorded using digital recorders and/or mobile phones and a rapporteur assigned to each table, taking detailed notes. The facilitators used an open-ended approach in ‘breakout groups’ which ensured the sessions were highly participatory as participants were encouraged to share their knowledge and experiences according to four thematic sessions, using a range of approaches, brainstorming and prioritisation/ranking exercises. All workshops ended with plenary sessions in which the various breakout groups presented their summarised ideas to the whole group, and the lead facilitator summarised the key discussion points. The workshops lasted on average 2.5 hours and the outcomes of the framing workshops informed the design of the TickSolve project priorities.

#### Key-informant interviews

We supplemented the MS workshops with a series of in-depth interviews (*n* = 18) conducted between November 2022 and February 2024 with key actors (directly/indirectly) influencing deer and woodland management across the study landscapes. In total, 35 potential participants were invited via email and 18 agreed based on availability. Interviewees included officials of The Deer Initiative Partnership, Woodland Trust, Royal Society for the Protection of Birds (RSPB) and landowners and were selected based on their experience, expertise and involvement in land management. A semi-structured interview guide (structured in 4 thematic sections – background of key-informants, knowledge of ticks and TBDs, deer/woodland management and prioritisation of interventions) was used and all interviews (see Additional file 1) were conducted on a one-to-one basis in English via telephone or zoom, according to interviewees’ preference. The interviews lasted on average 45 min and were recorded with the prior-informed consent of participants.

#### Data analysis

The collated qualitative data (from workshops and key-informant interviews) were transcribed and thematically analysed using NVivo (version 20, QSR International), using a combination of deductive and inductive approaches, with the individual as the unit of analysis. Three of the authors (FAA, EST, HK), one of whom did not participate in the workshops (HK), independently read, transcribed and coded the anonymised transcripts. We first coded the transcripts based on the workshop facilitator guide ((i) knowledge and perceptions about ticks and tick-borne diseases, (ii) perceptions of deer, movement and management, and (iii) prioritising options for woodland/deer management). Workshop data were synthesised through thematic mapping to identify gaps in the information gathered in the workshops [44]. Preliminary coding was validated using the first focus group transcript (New Forest workshop 1) and followed by a collective discussion by the team. Through this iterative process, emergent codes (and sub-codes) were then organised into themes and sub-themes. The remaining transcripts (i.e. for New Forest and Aberdeenshire workshops) were then coded and the emergent themes defined and finalised. Based on self-reported scores (using coloured dot voting from 1(limited knowledge) to 3 (high knowledge)) participants’ level of knowledge on ticks and TBD risks were assessed. The workshop data were triangulated with the interview data based on which inferences and conclusions were drawn [45]. Exemplar quotations captured in the ensuing paragraphs represent the final themes and divergent viewpoints expressed by study participants.

## Results

The workshops were highly participatory as all participants actively engaged in the discussions, exemplifying their level of knowledge and/or interest in the subject matter. While most participants concurred that risks associated with ticks are an important public health concern that needs to be proactively addressed, there was some differentiation regarding the contextual feasibility and potential uptake of the identified environmental interventions in the study landscapes. The key themes are summarised in Table 1.

**Table 1:** Summary of key themes and sub-themes from the thematic analysis

Main theme	Sub-themes
Participant tick and TBD knowledge	• Awareness of tick and tick-borne diseases (biology/life cycle of ticks, disease transmission pathways, at risk populations)
	• Extent of tick population and infection in people
	• Common tick-borne diseases
	• Tick hotspots
	• Personal protective measures
	• Usual sources of information
Risk perceptions	• Severity of TBDs – extent of concern about ticks and TBDs
	• Perception about tick populations (seasonality of ticks)
	• Key challenges for tick and TBD management
	• Responsibility for tick management
Deer management	• Deer and wildlife population dynamics
	• Deer movement patterns
	• Deer-tick associations
	• Current management strategies
	• Challenges for deer management
	• What would deer management success look like
Prioritisation of environmental options for deer, wildlife or tick management	• Other initiatives that have worked well in dealing with tick and TBDs
	• Potential measures and strategies to control ticks
	• Acceptability and perceived effectiveness of different environmental interventions
	• Agri-environmental schemes and tick dynamics

### Awareness and risk perceptions of ticks and tick-borne diseases

**Fig. 2:**
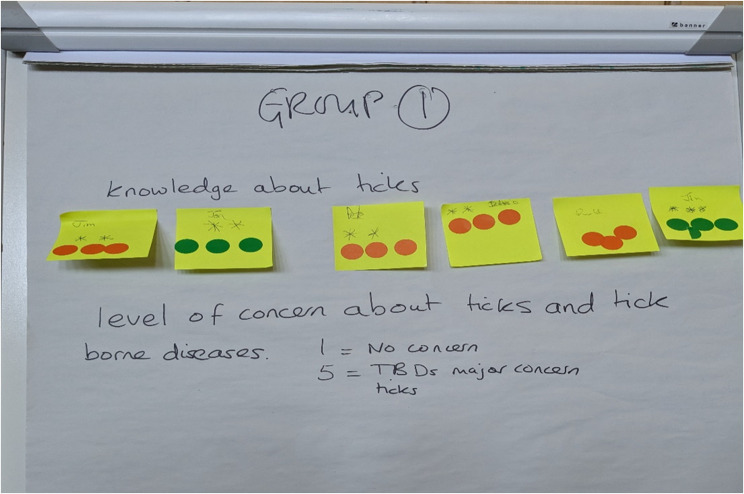
Example of group rating exercise conducted during the workshops

While there was variation in participants degree of knowledge, general awareness of tick and tick hotspots was high and broadly consistent across the three workshops (see Fig. 2). To the extent that the sample recruited were all involved land, forest and wildlife management (e.g. land managers, foresters, gamekeepers) in some respect, they had had firsthand experience and/or tick encounters (as part of their day-to-day operations) which could have informed their level of awareness and perhaps concern about the tick associated health risks. Two typical views expressed by a head ranger and landowner in separate workshop discussions are illuminating about the need to raise awareness among staff involved in land management and general practitioners involved in treating TBDs:

*“Several of my rangers have contracted Lyme disease. I’ve always encouraged my staff to speak to their GPs [about Lyme disease] and make sure they are carrying tick card that say ticks are here….”* (W1- ranger, New Forest).

*“Local GP surgeries can have low awareness of ticks and tick-borne diseases*,* people with infection can be dismissed*,* and under diagnosed or diagnosed at A&E [accidents and emergencies]*,* very dependent on GPs personal interest.”* (W2-landowner, New Forest).

**Fig. 3:**
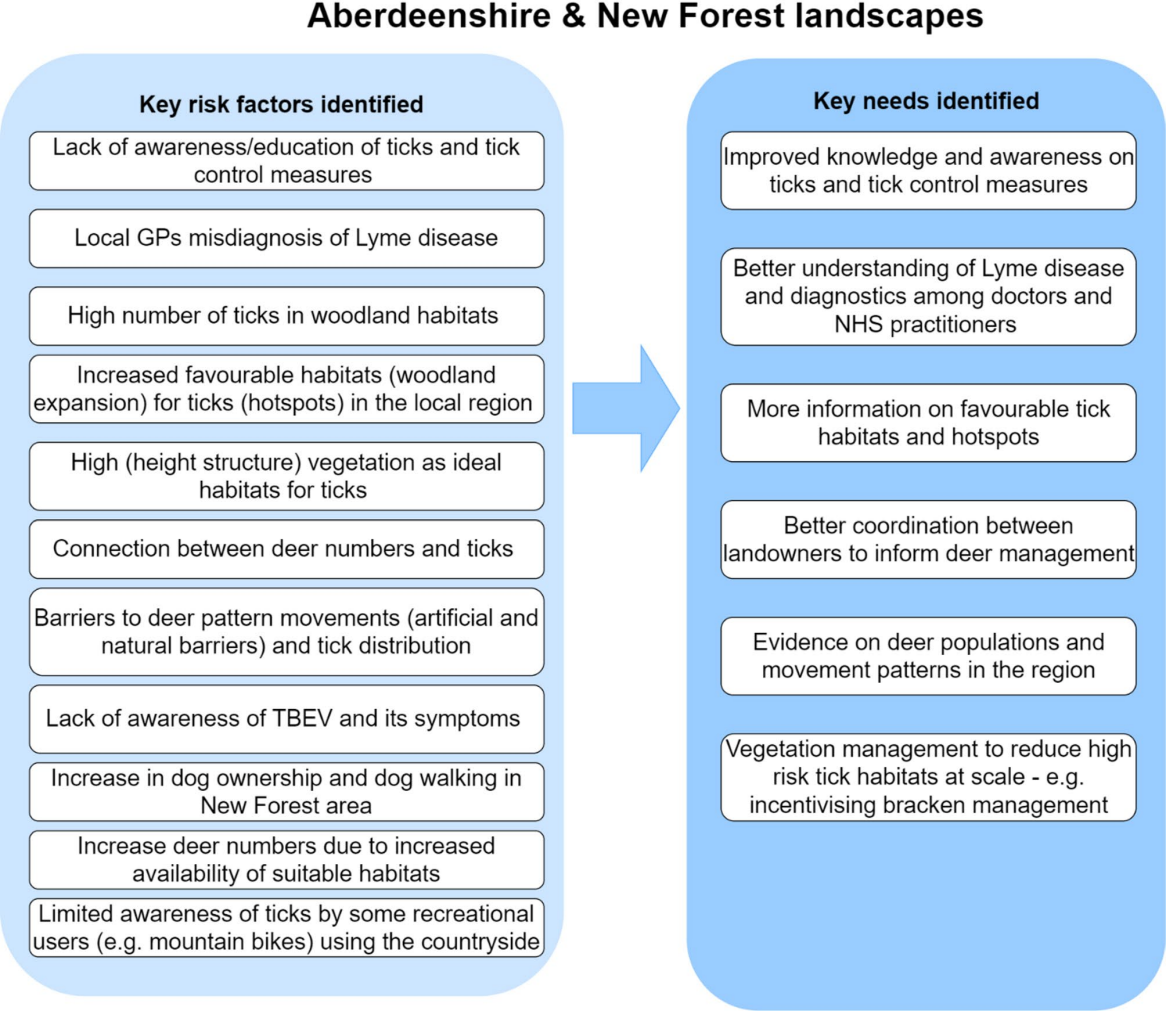
A summary of the key perceived risk factors and needs mapped during the three stakeholder workshops

Nuancing the narrative, a cross-section of the New Forest workshop participants argued that concerns lie mainly with visitors who are not as aware of ticks and TBD risk in the region and are therefore at the worst risk due to lower likelihood of taking personal protective measures. Indeed participants generally expressed that recreationists are more aware of other dangers, such as adders, than they are of ticks. As one participant (from the New Forest verderer community) explains, “*it is mostly the long-term New Forest residents and the planning community who are aware of ticks*,* while other locals who live in the forest area are generally unaware…*” (W2-New Forest). Corroborating this assertion, a land policy actor in a separate interview asserted: “*To me*,* I don’t think it’s about the changes in the land management practices* [*i.e. to reduce risk of human-tick encounters*], *but it’s more about human behaviours and creating awareness of diseases and the risk of ticks”* (KI004). Across the three workshops the consensus was that risks from ticks/tick-borne diseases should be featured in the training of doctors (and part of continuous professional development (CPD) trainings) as local GPs tended to struggle with the diagnoses of Lyme disease, and overall were not referring people for testing (Fig. 3), even though this is a national hotspot for Lyme disease cases (see Fig. 1). Key perceived at-risk occupational groups identified by participants included foresters, deer managers, gamekeepers and horticulturalists.

On the question of hotspots of ticks, a recurring theme from the discussion was that wooded areas have higher densities of ticks than open areas. High vegetation, bracken, conifers, purple moor grass and cereal crops were particularly highlighted by participants as ideal habitat for ticks. Participants also asserted that the risk of ticks seems lower around heathland/acid grassland with heavy grazing levels due to low sward height, which contradicts extant ecological evidence suggesting higher densities of *I. ricinus* ticks in ground vegetation, particularly lowland livestock pastures [46]. Adjacent bracken that are lay ups (resting places) for deer were perceived to have more ticks and fronds to be the right height for questing ticks to brush onto people. The perceived high-risk habitats have all been associated with *Ixodes ricinus* in empirical ecological surveys except cereal crops, though the shrub/hedge ecotones between forest and arable land can support tick populations [47, 48]. A participant recounted that it had been previously assumed that ticks were more abundant during spring/summer, but they have been finding ticks all year round on themselves and on their companion dogs. The awareness of ticks and tick hotpots amongst workshop participants therefore seem to be influenced by the frequency of their encounters with ticks – although it was conceded that certain hotspots might be due to how well ticks are visible (when attached to a host and engorged) rather than the actual extent of tick population in the area – along with being or knowing someone being infected by tick-borne disease. When asked about their existing sources of tick-related information, most participants had multiple sources of information, with the UK Health Security Agency (UKHSA), National Health Service (NHS) and family and friends mentioned as the topmost sources. Altogether, the discussions indicate that risk of ticks is perceived to be strongly correlated with vegetation type and structure rather than broad habitat types perhaps conditioned by the fact that the workshops were focussed on woodlands and woodland users.

### Deer, movement and management – seeing the bigger picture

Deer species are not considered competent transmission hosts for Borrelia spp. that cause Lyme Disease (since deer sera lyses some strains of Borrelia [49] or for the virus that causes Tick-borne encephalitis). However, deer are reproductive hosts for ticks since they feed adult females and thus play a key role in maintaining tick populations and increasing TBD risk in different parts of Europe [21, 50, 51]. To the extent that different deer management strategies have differing implications for tick life cycles and associated risks [21], it was instructive to explore with stakeholders their perceptions of deer (as ticks reproductive hosts) and current management and the impact on disease risk transmission at the landscape level. In this context, participants were first asked about their general assessment of deer population in their local area, and their viewpoints used to situate the discussions that followed. Across the three workshops, participants demonstrated extensive knowledge regarding deer, their habitat use and movement including variation in these behavioural attributes between species. Of the five species present in the New Forest (fallow, roe, red, sika and muntjac), fallow deer populations were perceived to have grown out of control and muntjac, an invasive species, are spreading in range; with muntjac known to be difficult species to manage. Deer herds (mainly fallow) were observed to be highly mobile in the New Forest area, making use of the connectivity between the open forest and adjacent areas such a cropland to move around and feed. They are also seen to shift between crown lands managed by Forestry England and adjacent private sites. A recurring theme from the discussions was that the local deer population is increasing, with fallow deer particularly highlighted, due to their “canny” behavioural avoidance of stalkers (e.g. can become nocturnal). In Aberdeenshire, participants likewise observed that local deer population have increased considerably and had become a key woodland management challenge, deeply rooted in complicated socio-cultural, political and economic arrangements, particularly in the Scottish Highlands. Two typical views expressed by a land manager gamekeeper in separate interviews are illuminating:

*“Deer populations have increased across a range [between] different species. Ranges have increased as well*,* so overall there’s more there in more of Scotland than possibly that ever has been. That’s not necessarily a problem everywhere*,* but it is in some places*,* and the only effective way of managing their populations is through lethal control.”* (KI013).

*“Obviously with the current trend of planting more trees…*,* deer management is higher up the woodland management agenda and being looked at more. So there’s a lot more pressure to kill more deer by the government*,* driven by the Forestry Commission.”* (KI016).

Across the three workshops, participants highlighted the absence of apex predators for deer, high fecundity and low mortality, Covid-19 and low culling rates as key drivers for the increased local deer population. Comparing the different deer species, deer and land managers particularly observed that roe and red deer were easier to manage through culling.

#### Dynamics of deer–tick associations

To further understand the interplay between deer (as reproductive hosts) and tick distribution on a landscape level, we asked participants about deer movement patterns (as a function of tick spread across different habitats) and related barriers in their local contexts. Overall, most participants (particularly landowners and deer managers) noted variations in tick loads in different deer species, with roe and muntjac perceived as “clean” and tending to have lower tick loads relative to sika and fallow. In a counter perspective, some participants expressed concern about the seeming over-emphasis on the role of deer (as implicated in the tick lifecycle) arguing that deer are part of the *“grazing herbivore”* problem not a *“deer problem”* (W2-New Forest). In this respect, one interviewee (a wildlife ecologist) underscored the need to rationalise the so-called *“deer problem”* as part of a broader spatial challenge of overlapping wildlife and human spaces:

*“The problem we have with wildlife (deer in this context) is not only when wild animals become very abundant*,* but it’s partly because most land in Britain is used by people*,* so it’s inevitable that as wildlife populations increase*,* then they start to have an impact…”* (KI003).

In the New Forest, several participants further highlighted an increase in livestock density in the area (from 7000 to about 20000) since environmental land management schemes was instigated in the region. Concerning deer movements, the consensus across the three workshops was that deer can navigate different habitats in the landscape and that there are not many barriers to their movement. Participants underscored the versatility of deer populations in their movement, noting that deer fences in the local area are ineffective and that deer are able to cross large roads and are even able to use the underpasses and also swim across rivers. Altogether, the discussions seemed to suggest that creating barriers to deer movement would be very complex and perhaps counterproductive given deer capability to cross such barriers, if present, and the fact that implementing barriers such as fences may simply push deer onto adjacent land.

#### Deer management and associated challenges

While there was a general agreement that deer-centred management is critical considering current developments around woodland expansion, participants equally highlighted the need to transition from a reactive to a more proactive management focus. Aberdeenshire and Wessex workshop participants argued that deer as ‘common pool’ resource has meant that a collective and/or joined-up rather individualised management approach is needed for meaningful impact at a landscape scale. Consistent with this observation, two interviewees separate conversations concluded that a holistic approach is needed, deer population control may not be necessary everywhere and needs to be contextualised such that management is tailored to local stakeholder priorities and other land management exigencies:

*“I think we need to try and foster an awareness that deer can’t be managed just by one woodland owner in isolation. Management needs to be done by a community and that community needs to understand the [deer] problem and accept the measures. It is not necessary to have low populations everywhere*,* but we can have some areas [with] high [deer] population so it’s all about accepting that management needs to be done to an extent in some areas and trying to meet the diversity of views”* (KI022).

*“The whole forestry system needs to be looked at; you know. So*,* for example*,* with managing deer*,* but not squirrels. A joined-up approach is needed”* (KI015).

Reflecting on the role of Deer Management Groups (DMGs) as convening platforms for collective decision-making, some participants expressed the need for caution about in-group politics since there is a potential risk that management priorities could be skewed in favour of influential actor groups. Participants pointed to the seeming tensions between bigger estates and the stalker and crofter communities, given their differing priorities and degree of influence in the current Scottish government deer management consultations. Reflections by a land manager and stalker community representative in separate interviews about the need to maintain a cautious approach to collaboration planning and expectation management are illustrative:

*“Deer Management Groups are critical but can also lead to conflicts. If one estate wants to reduce [deer] numbers to increase trees*,* another estate wants to have higher deer numbers for sporting reasons. The deer move between the two estates*,* so that can cause conflicts between neighbouring estates as it were. It’s a very simplified version of what can happen. It’s a lot more subtle than that…”* (KI017).

*“Deer management is a very cultural thing*,* and each country approaches it in a different way. In some contexts*,* hunting is very popular and there is a large body of hunting people wanting to do hunting*,* and so inevitably*,* that becomes a powerful influence on how it’s [deer management] is done and how it is approached.”* (KI009).

In discussing current deer management challenges, bringing venison into the food supply chain through a game dealer and fencing off more targeted areas that deer roam around and out of plantations - especially in areas of the forest where woodland regeneration is occurring - were recurring themes. Across the three workshops, participants noted that deer management was not particularly profitable especially as venison is still marketed as an expensive/luxury meat. One participant had mentioned that exclusion fencing has allowed flora regeneration, and the exclusion of deer had meant a drop in ticks as well. However, one of the challenges is that financial and institutional capacity are needed to install and maintain the fences in the long term and ensure it is effective. This points to the wider challenge with regards to the resources required for managing deer – especially for conservation organisations – with the financial and material resources required for keeping the deer population under control being significant. Other challenges have included the increasing interface between wildlife and urban areas – with new urban developments overlapping wildlife and deer population areas, along with the lack of coordination between different landowners/organisations in dealing with deer and ticks on a larger scale.

The lack of provisions for deer management in agri-environmental schemes was mentioned as another key challenge. Several participants observed that the lack of provisions for exclusion fencing or fatal deer control, despite the growing focus of schemes such as the Environmental Land Management schemes (ELMs) have on connecting and restoring woodland habitats along with tree planting. There are concerns that the growing shift towards increasing connectivity and biodiversity enhancement, which might create the right conditions for tick growth and increased deer movement, without any provisions from ELMs, will further add to the challenge of management. One participant did note receiving funding for deer control from a charity, however, this reiterates that requirement for deer control should be assessed on a national level. Two typical views expressed by workshop participants reflecting on the need for inclusion of deer management subsidies in existing agri-environmental schemes are illustrative:

*“Most policies are currently not thinking about ticks*,* good to add it in there. We are in the middle of a nature and climate emergency but can’t claim for deer management even though it is clearly adversely affecting biodiversity and many of [the] deer are non-native species. New hedges and saplings planted are eaten by deer so on back of woodland expansion policy*,* could have subsidies for deer management”* (Workshop-2, New Forest).

*“Not natural control of deer*,* will hamper biodiversity net gain*,* will increase ticks. Important for government to fund control of deer populations*,* to reduce tick populations. What about cattle and sheep supporting tick populations instead – will these decline over time or not as we seek to be more self-sufficient in food*,* and reduce importation?”* (Workshop 3, Aberdeenshire).

### Prioritization of environment-based interventions

Across the three workshops, several environmental interventions ranging from habitat (e.g. vegetation management/landscaping, application of acaricides) to deer-centred interventions (e.g. deer fencing, deer culling) were identified (see supplementary Figs. 5 & 6). Participants particularly highlighted the importance of focusing on interventions centred on deer management rather than tick management, as the latter was peripheral to their current land management priorities. In endorsing deer removal as the preferred intervention, a deer policy actor explained: “*well*,* it’s the only strategy… to manage the populations is to control them with lethal control. There are no natural predators.”* (KI019). Overall, participants highlighted there is no single one size-fits all intervention for deer and/or tick management, noting that a combination of interventions are needed to reconcile differing stakeholder priorities and expectations about land management on a landscape scale (see Table 2).

**Table 2:** Summary of discussions on potential environmental options, challenges and opportunities for implementation

Ranking	Intervention	Challenges	Opportunities
1	Education/awareness campaigns	• Entrenched practices/behaviour and distrust among local stakeholder groups	• Leverage existing regional networks of national park management and landowners to disseminate messages to the public (including recreational users)
			• TBD awareness raising highly prioritised and less expensive
2	Deer culling (removal)	• Difficulty in obtaining data about deer numbers.	• Experienced and knowledgeable local stalkers
		• Uncertainty of the acceptable level of cull	• Financial incentives for business
		• Not suitable for small-scale landowners	• Widely perceived as a management priority
		• Insufficient information on effectiveness in terms of reducing human tick exposure.	
		• Incompatibility with land management objectives (e.g. high deer numbers for wildlife tourism versus deer impacts on habitat connectivity and increased woodland cover)	
		• Disturbance of stalking effort by the public (e.g. recreational woodland users)	
		• Limited market and opportunities for venison (low public consumption)	
		• Ethical concerns by people	
3	Deer fencing	• Initial and maintenance cost	• Quite successful in protecting vegetation (stands of saplings)
		• Damaging to wildlife and habitats	
		• Opposition from recreational users (e.g. walkers)	
		• Political sensitivities	
		• Roe deer particularly efficient in breaking through.	
		• Ineffective in the long-term as become increasing ‘porous’.	
		• Concentrates deer density and intensifies their impacts (e.g. over-browsing) outside the fenced areas.	
		• Perceived as not likely to influence tick density.	
		• Visually obstructive	
4	Landscaping/vegetation management	• Not feasible on a landscape scale due to limited resources	• Opportunity to select plants not favoured/palatable to deer.
		• Difficult to ascertain the spatial extent of tick reduce high-risk tick habitats.	• Reduces human tick encounters in high-risk tick habitats
		• Not good for biodiversity conservation and potential negative trade-offs – e.g. for pollinators	
5	Immuno-contraception vaccines/fertility control for wildlife (recommended for deer hosts)	• Not enough information regarding feasibility on a landscape scale	• Ethically safe
		• Unwillingness of landowners to bear cost of immuno-contraception.	• Positive for deer welfare
		• Entrenched views on traditional deer management	
6	Topical acaricides for propagation hosts (deer)	• Pesticide exposure in deer species	• Ethically safe
		• Unwillingness of landowners to bear cost of treatment with acaricides.	• Positive for deer welfare
		• Uncertainty of efficacy on a landscape scale.	
7	Deer self-treatment bait (acaricides) devices	• Potential negative impact on wildlife food chain	• Ethically safe
		• Pesticide exposure in deer and other non-target wildlife species	• Positive for deer welfare
		• Limited information about usefulness and effectiveness.	
		• Lack of cooperation of landowners	

While participants generally acknowledged the importance of targeting interventions at landscape scale to be impactful, the majority argued that prioritizing public and GP sensitization campaigns, particularly around risks from ticks, hotspots areas/habitats and personal protective/preventive measures, as opposed to solely focusing on environmental control measures could be better in reducing risk of human exposure to ticks, at least from a land management and public health security perspective (see Table 2 below). In this sense, participants expressed mixed views in terms of the feasibility and acceptability of suggested environmental interventions (e.g. deer fencing, deer culling and vegetation control/landscaping), indicating which ones they considered either not appropriate or not a priority in this context. For instance, deer exclusion and removal strategies (e.g. deer culling) were deemed to sometimes be at odds with land management priorities (e.g. wildlife tourism) and there was perceived to be limited evidence on their effectiveness in the study regions. In both the Aberdeenshire and New Forest workshops, some participants observed that deer exclusion (e.g. deer fencing) and removal (deer culling) strategies have the propensity to confine deer to specific locations, reducing species richness (i.e. drastically reduce the population of some species, particularly roe and red deer perceived as easier to manage relative to fallow, sika and muntjac) and incentivize deer shooting as a hobby.

“*“The national picture is to reduce the muntjac or fallow numbers but at the moment the venison price is low*,* and we only shoot for the freezer and leave the rest. You will have to build and incentivise the market so that it can absorb more venison. I think that putting pressure on landowners will be met with resistance and there are sometimes good reasons for that. Whether they want to shoot deer on their land or don’t want to*,* that’s entirely up to them. You know*,* landowners are running a business…”* (KI021).

Vegetation control/landscaping was seen as prohibitively expensive and not feasible at landscape scale, but participants highlighted same as a preferable option for biodiversity conservation. Erecting deer fencing as a means of excluding deer and other ungulates on a landscape scale was considered unsuitable, particularly by landowners and managers as fencing out wildlife sometimes conflicted with their land management priorities [52]. In instances where deer fencing has been implemented in the New Forest, for example, participants commonly argued that deer exclusion in one part of the landscape simply diverts and increases deer impacts in adjacent parts of the landscape (i.e. more accessible areas with suitable forage). In any event, the canny nature of deer has meant that they are able to circumvent fencing as a movement barrier, thus fencing is altogether ineffective and expensive to maintain in the long-term. A typical view expressed by an interviewee (landowner) when asked about the suitability of deer fencing at scale are particularly illuminating:

*“Well*,* in woodlands if fencing is used to protect young trees from browsing that works up to a point. It’s more expensive to put fencing around the young trees than it is to plant trees themselves*,* so it’s the most expensive part of tree regeneration and it’s a bit unsightly. It is not good for people who want walk in the woodlands.”* (KI018).

In discussing public education campaigns (targeting all local stakeholder groups, including landowners, foresters, and recreational users) as a preferred intervention strategy, workshop participants underscored that clear messaging for visitors to the New Forest, for example, is vitally important in raising awareness. Places suggested as ideal for posting such information are in guest accommodations, campsites, local businesses, and at veterinary practices. Of equal importance is ensuring the information is continuously updated and accurate and how the message is presented. In justifying the rationale for clear and balanced messaging, a conservation manager reflected, “w*e are not just a conservation organisation. We’re also an access charity*,* and we’re very much about getting people outdoors and close to nature which might sound ominous when we’re talking about ticks. But uh*,* today*,* in general terms*,* it’s lovely to make sure we’re encouraging people to go and experience the outdoors…”* (KI012).

Several participants expressed the need to avoid negative tone and presenting the benefits of managing the risks, as opposed to the dangers. Some participants further noted the importance of balancing the amount of messaging being presented, as too many signs or messaging might lead to the public being less likely to respond to them, an issue participant pointed out as “sign blindness”. This observation lends credence to Slunge & Boman’s [53] and Quine et al. [54] argument that the challenge in tick-related risk communication is how to encourage precaution without causing alarm, risking deterrence from outdoor recreational activities, which may have associated health benefits. In negotiating a ‘middle-ground’ solution to the risk communication quandary, one Aberdeenshire workshop participant suggested, *“it probably comes targeted information on risks from ticks and personal protective measures*,* for example tucking your socks into your wellies*,* and just getting out there and checking yourself*,* thoroughly*,* I think. You know*,* three million people walk my countryside every year. How many of them come away with a tick? I have absolutely no idea! Not a job of I wouldn’t even know how to guess…”* (Workshop 3, Aberdeenshire).

Altogether, the foregoing gives the indication that contextualization of a combination of priority interventions may be needed, necessitating concerted engagement of all stakeholder groups to reconcile their priorities and expectations regarding the uptake of interventions aimed at reducing tick-related public and animal health risks at the landscape scale.

## Discussion

As tick-borne disease burdens continue to evolve and expand across Europe and the UK [3–5], particularly in view of climate change and woodland expansion policies [10, 27, 55, 56], there are growing calls for concerted action across science and policy spheres to manage TBD risks as an emerging “public-bad” problem [57]. Recent studies have advocated land management as a plausible ‘landscape’ tool to help address public and animal health risks associated with land use change [1, 4, 24]. Nevertheless, land management decision-making is not neutral but often characterised by conflicting priorities and/or contestations, with far-reaching socio-economic, ecological and political ramifications necessitating the importance of eliciting different stakeholder perspectives and priorities in land management decision-making processes [20, 23, 58]. In our study, we explored stakeholders’ risk perceptions, knowledge, and environment-based tick control options in the context of woodland expansion and agri-environmental schemes (AES) in two mixed woodland-farmland landscapes in the UK known as historical hotspots of Lyme Disease.

Our results show a high level of awareness and knowledge regarding ticks and tick-related health risks in the study regions, with known *Ixodes ricinus* habitats of woodland edges, bracken and long grasses as particularly identified “hotspots” harbouring high tick densities. This finding seems to contradict prior participatory research [27, 58], which reported limited spatial knowledge of tick densities and locations among laypeople in mainland Europe. Inconsistency in results is likely attributable to differences in study populations, disease history, and socio-ecological context, including health and land management systems. Though scant long-term monitoring of tick populations and distribution is available, the consensus notion that the distribution of ticks is expanding across the study landscapes is also broadly consistent with an annual increase of 5–6% in the England and Wales locations from which people are reporting tick bites to the UK Health Security Agency [59, 60] as well as with studies of stakeholder perceptions and practices elsewhere in Europe [27, 58]. The association of high tick densities with specific habitat types, not only demonstrates a good spatial awareness of tick locations among land and wildlife managers but aligns with the argument that tick-related risks are highly context-dependent and not evenly distributed across the landscape [21, 58, 61, 62]. This illustrates the importance of place-based approaches to better understand how people’s risk perceptions and habitat use maps onto landscape level tick-host-pathogen density dynamics that underpin exposure risk, to inform co-designed, evidence-based interventions that are contextually relevant and appropriate [1, 20, 58, 63].

Although perceived risk of tick-bite exposure among study participants was high, corresponding with earlier research [53, 58], ‘indirect’ deer-centred interventions (e.g. deer removal) were commonly preferred to more ‘direct’ tick control management (e.g. landscaping, bracken control, acaricidal measures) to minimise risk of human-tick encounters at scale. This finding is not entirely surprising and can be attributed to a number of factors. One possible explanation for the stated preference for deer-centred interventions is that risk perceptions can be dampened as people get used to living with the risk of tick-bite exposure and TBDs and perhaps perceive ticks as less serious management concerns relative to increasing wild deer populations or other health issues for themselves or livestock [23, 53]. The fervent debate around the Deer Management Nature Restoration Orders (DMNROs) in Scotland perhaps lends some empirical credence to this explanation [64–66]. A second explanation may be that the cost of implementing tick control management (particularly in endemic areas with high tick densities) at scale is perceived to be greater than the supposed and uncertain benefits. This assertion is also supported by the skepticism about the perceived effectiveness of tick control management and concern regarding environmental contamination and potential negative trade-offs for biodiversity (e.g. off-target effects of acaricides, acaricide resistance in tick population) and land management [22, 53, 67]. Prior studies in other contexts have reported on some perceived costs of tick control management including environmental risks from use of acaricidal measures and informational costs (lack of implementation know-how) [22, 53, 63, 67]. Aenishaenslin et al. [68] for instance, reported that Swiss stakeholders involved in decision-making for Lyme disease preventive strategies were less inclined to include some tick control interventions (such as application of acaricides or landscaping) at large scale as hypothetical interventions in decision-making due to their potential environmental impact being at odds with the values of the wider populace.

While it is difficult to directly compare different empirical studies due to differences in context and/or methodologies, some experimental studies have been conducted in highly (TBD) endemic regions elsewhere (mostly in the United States) to assess the effectiveness of different ecological interventions in reducing tick abundance in forest areas. Overall, these studies have shown mixed results in terms of effectiveness of assessed interventions and are altogether inconclusive [25, 53, 69]. A recent US study on effects of prescribed fire on tick spread and propagation by Fulk et al. [70] demonstrated that while *Ixodes scapularis* ticks can recover rapidly following a burn, yearly, high-intensity prescribed burns can reduce the prevalence of ticks in and around the burned area. The authors further reported that frequent burning can slow establishment significantly. In their experimental study, Keesing et al. [69] reported tick control system (TCS) bait boxes (with acaricide fipronil [71]) that attracts small mammal hosts reduced abundance of questing nymphal black-legged ticks in forest habitats by 53% compared with placebo controls. Abundance of ticks attached to small mammals also reduced by ~ 50%, whereas the deer intervention, Met52 fungal spray, was not associated with significant reductions. These few extant studies on effectiveness of environment-based interventions to control TBDs often fail to systematically evaluate the trade-offs between these interventions and biodiversity and land management across different socio-ecological contexts [1, 24]. The dearth of available information suggests that empirical research is needed on the safety, effectiveness and cost-effectiveness of environment-based tick control measures on a landscape scale for evidence-informed decision-making across geographic regions [20, 59, 72].

Consistent with the findings of earlier knowledge, attitudes and practice (KAP) studies [27, 53, 67], our research suggest strong local support for targeted information campaigns and educational interventions on tick-bite and TBD prevention, and highlights a seeming disconnect (in terms of information access and sharing) between at-risk groups and the scientific and medical actors (i.e. GPs and other NHS practitioners) in the study regions. To the extent that there currently exist limited avenues for accessing credible tick-related information in the study contexts (from the perspective of woodland managers) perhaps lends credence to this assertion. Indeed, the endorsement of risk communication strategies as the most preferable preventative strategy is not entirely surprising considering the concern about unknown environmental impacts and perceived ineffectiveness of tick control management at landscape scale (Sect. 3.4). Nevertheless, the effectiveness of educational interventions such as large-scale communication campaigns in fostering changes in TBD preventive behaviours within high-risk populations is questionable and at best very context-dependent [53, 54, 67, 73]. Regardless, only a few studies have reported on the effect of educational interventions in The Netherlands and in the United States [26, 74]. Shultz [75] argued that the concern for environmental issues (in this context tick-related risks) is not necessarily reflected in peoples’ pro-environmental behaviours, but rather highlights an underlying impression of their sense of place and/or attachment with the natural environment. As evidenced in Sect. 3.4, most participants tended to take a broader viewpoint of the beneficence of the natural environment (as functional spaces for diverse occupational and recreational groups) highlighting the need for contextualised, balanced and targeted risk messaging that is accessible by all at-risk groups. This observation aligns with calls for refining extant risk communication approaches via perhaps alternative educational tools, including school-based interventions, the use of video games and mobile phone applications, which offer low-cost and accessible models for improving risk communication on tick-bites and TBDs in endemic regions [67, 76–78]. As highlighted in the literature, which our findings support, the dissemination of trustworthy and credible information should be at the heart of such innovative risk communication tools. While the UKHSA and NHS were identified as principal TBD information sources, participants equally mentioned tick-related information was sourced through social networks (e.g. family and friends, social media) which can be unreliable and risk spreading misinformation [53]. This suggests a need for concerted engagement between decision-makers and stakeholders in woodland management (e.g. woodland owners and managers, deer managers and foresters) in understanding the barriers and opportunities for effective risk communication and knowledge exchange [25, 63, 68]. This further warrants attention by researchers and decision-makers alike to the often “epistemological anxiety” (i.e. tendency for actors’ knowledge to be shaped by their stake in a context) and/or value judgement about local knowledge to optimise such “practical” knowledge forms in decision-making [27, 79]. Sylvain et al. [27] explained that such ‘knowledge hybridisation’ in the decision-making process helps avoid selective knowledge usage and opens the window for knowledge exchange and (in-)validating certain information and/or management practices.

These findings have implications for policy direction on land use and TBD governance in woodland landscapes, especially UK’s post-Brexit implementation of Environmental Land Management (ELM) system [15, 80, 81]. Our study for instance revealed that deer management appears to be overlooked and does not feature in extant policy on AES despite their potential link to the increase tick activity and associated disease risks, particularly Lyme disease [4, 46, 56]. This lacuna in extant woodland expansion policies has re-ignited policy discussions on wild deer-woodland governance in the UK, particularly in Scotland [81, 82]. Indeed, some studies have demonstrated an association between deer abundance and tick-borne pathogen prevalence and Lyme disease hazard in woodland areas [83, 84]. To help address the likely increase in risk of human-tick encounters and related TBDs linked to increased woodland cover and green spaces [24, 85], it is vitally important that new woodland expansion and ELM initiatives account for tick and TBD risk assessment and associated mitigation options in their implementation processes. In this respect, the Scottish government efforts [64] to broaden stakeholder consultation, are encouraging and could be leveraged as an engagement outlet to reach out to other stakeholders at the fringes of woodland governance.

This study has some limitations that might affect the interpretation and generalisability of the findings. First, in terms of stakeholder representation, land and deer managers and foresters were particularly over-represented at the workshops and our cohort may not be representative of the sampled population, including for example recreational groups that are remote from woodlands or use them less frequently. Since we specifically targeted land stakeholders involved in and/or affected by land use governance (informed by the TickSolve project objectives) and perceived as key at-risk groups in the focal landscapes, it implies that the said cohort of stakeholders were more likely to enrol and participate in open communication about “public bads” (in this context ticks and tick-borne diseases posing a significant societal problem adversely impacting health and well-being of at-risk populations) [63]. To extent that this assertion holds true, then it can be assumed that the risk-populations (based on the likelihood of tick exposure) were not underrepresented at the workshops. Moreover, the New Forest workshop witnessed a higher participation relative to the Aberdeenshire one, partly due to the facilitated access to the large pool of the NFPA stakeholder network in the New Forest region who were willing to engage in open communication about the subject-matter. The limited stakeholder representation at the Aberdeenshire workshop may be partly attributed to several reasons including for example, the timing and prevailing local tensions between different interest groups around deer management issues in the region. It is noteworthy that, at the time of the workshop organisation, the political stakes and local sensitivities around the deer question were high which perhaps affected overall participation. Besides, from the grapevine (at the time of workshop organisation), we gathered that it could be challenging to convene a multi-land stakeholder meeting due to access challenges and the risk of fuelling further tensions around the deer question, particularly among opposing factions. Resorting thus to a virtual workshop (based on participant preference) with a smaller number of participants afforded the space for ‘managed’ discussion and expectation management on the subject-matter. Furthermore, the supplementary (one-to-one) key-informant interviews thus afforded a more neutral space and ensured that the views of underrepresented actor groups (e.g. crofters and stalking communities) were sufficiently captured. A further consideration is the predominance of deer-centred discussions relative to the other thematic areas of interest, which might not necessarily imply less receptivity to the other habitat-oriented interventions but rather more familiarity with the former. This aligns with the argument that stakeholders often tend to gravitate towards topics of perceived importance and/or interests based on their contexts and experiences [63, 68]. The foregoing highlights the importance of reflexive thinking on researcher positionality and contextual sensitivity in stakeholder engagement. It further echoes the value of place-informed stakeholder assessment in ensuring broad and inclusive representation of key actors in the stakeholder engagement process.

## Conclusion

The management of ticks and TBDs as an emerging “public bad” and collective action problem is complex and challenging, necessitating a place-based approach that is anchored in a concerted cross-sectoral collaboration between various governmental bodies and at-risk communities to facilitate information and knowledge exchange and co-development of contextually relevant interventions and evidence. In keeping with the broader literature, our research suggest that while environmental tick control management may hold promise in minimising pathogen prevalence and exposure at the landscape level [86], there is the need for contextualised, bottom-up engagement (that accounts for potential trade-offs) to ensure proposed interventions align with stakeholder priorities and can be widely adopted in woodland management decision-making processes. This warrants additional empirical studies on the complex interplay between stakeholder characteristics, land-use practices, and perceptions about tick control management [22, 68]. Overall, our research suggests that singular interventions (aimed at addressing the ticks and TBD conundrum) will not work in isolation and that a blend of complementary strategies that are sensitive to different stakeholder needs and priorities may increase the propensity of effective collective organising and action in the face of a seemingly complex “public bad” phenomenon.

## Supplementary Information

Below is the link to the electronic supplementary material.

Supplementary Material 1

Supplementary Material 2

Supplementary Material 3

Supplementary Material 4

## Data Availability

Given the richness of the qualitative data and the potential for identifying individual human participants and violating confidentiality of the key informants who participated in the semi-structured interviews, our study dataset will not be shared openly but may be available upon specific request. Researchers wishing to access the dataset used in this study should contact the UK Centre for Ecology & Hydrology Institutional Data Access contact via Dr Festus Asaaga (email: fesasa@ceh.ac.uk).

## Abbreviations

AES: Agri-environmental schemes
ELM: Environmental land management
CPD: Continuous professional development
DMNRO: Deer Management Nature Restoration Orders
DWG: Deer Working Group
GP: General practice
KAP: Knowledge, attitudes and perceptions
LD: Lyme disease
MS: Multi-stakeholder
TBDs: Tick-borne diseases
TBE: Tick-borne encephalitis
TBEV: Tick-borne encephalitis virus
TCS: Tick control system
NGT: Nominal group technique
NHS: National Health Service
NFPA: New Forest National Park Authority
RSPB: Royal Society for the Protection of Birds
UK: United Kingdom
UKHSA: UK Health Security Agency
