# Supplementary material for: Exploring stakeholder perceptions and priorities related to reducing tick-related public health risks in natural environments of the United Kingdom
Source: BMC Public Health. 2025 Oct 2;25:3300. doi: 10.1186/s12889-025-24500-7 (PMC12492900; doi:10.1186/s12889-025-24500-7)
Supplement: Supplementary file 1 — Supplementary Material 1 [file 12889_2025_24500_MOESM1_ESM.docx]

**New Forest/Wessex Framing Workshop Agenda**

Monday, 14^th^ of November 2022

Avonway Community Center, Fordingbridge, SP6 1JF

| **1:00-1:45** | **Set-up** |
| --- | --- |
| **1:45-2:15** | **Registration + welcome (questionnaire)** |
| **2:15-2:30** | **Project overview** |
|  | **Workshop aims** |
| **2:30-2:55** | **PART 1: General knowledge and perceptions about ticks and tick-borne diseases** |
| **2:55-3:10** | **Break: Tea/coffee available** |
| **3:10-3:50** | **PART 2: Perceptions about deer/wildlife, movement and management** |
| **3:50-4:30** | **PART 3: Prioritising options for deer/wildlife/ woodland management** |
| **4:30-4:35** | **Break: Tea/coffee available** |
| **4:35-5:00** | **Project updates & Lyme disease talk** |
|  | **UKHSA Clinical Q&A** |
|  | **Closing comments and** **Thank you** |
