# Supplementary material for: Exploring stakeholder perceptions and priorities related to reducing tick-related public health risks in natural environments of the United Kingdom
Source: BMC Public Health. 2025 Oct 2;25:3300. doi: 10.1186/s12889-025-24500-7 (PMC12492900; doi:10.1186/s12889-025-24500-7)
Supplement: Supplementary file 2 — Supplementary Material 2 [file 12889_2025_24500_MOESM2_ESM.docx]

| **AGENDA FOR ABERDENSHIRE TICKSOLVE WORKSHOP** | |
| --- | --- |
| **9:20-9:30**  **(10 mins)** | **Team briefing** |
| **9:30- 9:40**  **(10 mins)** | **Registration + welcome** |
| **9:40-9:50**  **(10 mins)** | **Project overview** |
|  | **Workshop aims** |
| **09:50-10:35**  **(45 mins)** | **PART 1: General knowledge and perceptions about ticks and tick-borne diseases** |
| **10:35-10:40**  **(5 mins)** | **Tea/Coffee break** |
| **10:40-11:25 (45mins)** | **PART 2: Perceptions about deer/wildlife, movement and management** |
| **11:25-11:30**  **(5 mins)** | **Tea/Coffee break** |
| **11:30-12:15pm**  **(45 mins)** | **PART 3: Prioritising options for deer/wildlife/ woodland management** |
| **12:15-12:20pm**  **5 mins** | **Tea/Coffee break** |
| **12:20-12:30pm**  **10 mins** | **Communication + Future Engagement** |
|  | **Closing comments and** **Thank you** |
