# Supplementary material for: Exploring stakeholder perceptions and priorities related to reducing tick-related public health risks in natural environments of the United Kingdom
Source: BMC Public Health. 2025 Oct 2;25:3300. doi: 10.1186/s12889-025-24500-7 (PMC12492900; doi:10.1186/s12889-025-24500-7)
Supplement: Supplementary file 3 — Supplementary Material 3 [file 12889_2025_24500_MOESM3_ESM.docx]

**TICKSOLVE: Environmental solutions to reduce the risk of current and future tick-borne zoonotic pathogens in the UK**

**Interview Guide for Key Informants**

Thank you for your time for this interview. We have a few questions about ticks and tick-borne disease (TBD) management in the UK. Within our project, we are seeking to better stakeholders’ experiences, needs and priorities concerning TBD management, and how we can better respond and prevent TBD risks such as Lyme disease and tick-borne encephalitis (TBE) among “at risk” populations. As someone who is involved/affected (directly or indirectly) by measures to prevent or control the spread of tick-borne diseases in the New Forest/Wessex area, your insights in this regard will be extremely valuable.

**General Information about interviewee**

1. Details about the person, their occupation, education background, geographical area/scale over which they operate (if applicable).
2. Could you tell us about experience and job as a forester/gamekeeper/land manager? (Probes: years of experience, how role affects/ affected by tick-borne disease management)

**Experiences and Perceptions about ticks and management strategies**

1. Can you tell us a little about what you know about ticks? How familiar are you with ticks? (Probes: Where do they live? How do they travel? What is their lifecycle? What diseases can they transmit?)
2. Do you consider ticks to be an issue in this area? Why do you say so? (Probes: increase/ decrease in ticks in the environment, increasing tick-bites, risk of disease-causing pathogen exposure)
3. How worried are your about ticks? (probe: level of worry/concern – 1 (not at all worried) and 5 (extremely worried))
4. What do you think is driving the population (increase/decrease) of ticks in this area? (Probes: climate change, overabundant deer (roe/red/fallow/muntjac), available tick habitats, increased rodent population, pets, livestock)
5. Do you think environmental change (i.e. change or disturbance of the environment as result of both natural processes and human influences) affects ticks and/ or occurrence of tick-borne diseases in humans? If so, how? If no, why not? (Probes: increase/decrease in ticks, habitat distribution, woodland creation, climate, risk of disease-causing pathogen exposure)
6. Do you think certain land management practices affect tick population/occurrence of tick-borne diseases (e.g. Lyme disease) in humans? If so, which practices and how? If no, why not? (probes: landscaping, vegetation removal, increase/decrease in ticks, risk of disease exposure)
7. Are there places that are hotspots for ticks in this area? (Probe: habitat types – (e.g. leaf piles and litter, high grass areas, wooded areas, overgrown shrubs), determinants of tick abundance)

**Experiences and Perceptions about Deer and Deer management strategies**

1. How would you describe the deer population in this area? (Probes: increasing/decreasing, deer species, which crops are important as a food source for deer, type of woodland deer prefer and possible variation between deer species) perceptions about deer movement patterns, any change in proximity to areas people use, homes, recreational areas, farms)
2. What do you think is driving the increase/decrease in deer numbers?
3. Are deer numbers currently being managed? If yes, how? If no, why not? (Probe: management measures employed, frequency of use, effectiveness)
4. Is there any connection between deer/gamebird and ticks? (Probe: do you find there are more or less ticks where gamebirds are released, change in the numbers of gamebirds released recently due to COVID/ EU exit, Avian flu )
5. Why do you think that? (Probe: ticks on the animal, role in tick/disease transmission, risk of disease-causing pathogen exposure)
6. How would you describe deer movement in this area? (Probe: variation in movement patterns of deer species, do deer prefer to spend more/less time in large or small woodland patches, deer crossing of open agricultural fields to access different woodland patches, changes in deer movement patterns, preferred movement routes)
7. Are there any barriers to deer/wildlife movement in this area? If so, please explain (Probe: landscape barriers – (e.g. fences, roads, railway, highways, rivers, canals), housing developments, agricultural fields, woodland connectivity)
8. How do you think these barriers affect deer/wildlife populations or tick densities? (Probe: deer/wildlife mortality,

**Perceptions about interventions to control ticks and tick-borne diseases**

1. Are there areas on your rural estate/farm where deer or other large ungulates are currently excluded? If so how and why the exclusion? (probe: rationale for exclusion – (e.g. protect crop or garden, tick avoidance, environmental conservation, reduce risk of disease transmission), type of exclusion – (e.g. deer fencing, deer culling), effectiveness)
2. Do you think there is a connection between deer exclusion and ticks? Is yes, how (please explain)? (Probes: changes in deer movements with deer/scaring measures, increase/decrease in ticks, decrease in tick-bites, reduced risk of disease transmission)
3. What do you think of the following environmental-based measures to control ticks and tick-borne diseases? (Probe: acceptability, viability – (logistically or economically), usefulness/effectiveness)
   1. deer fencing
   2. deer culling/ exclusion/scaring
   3. spraying pesticides (acaricides) on livestock/ sheep mopping
   4. application of acaricides to deer
   5. cutting vegetation short alongside trails
4. In your opinion, are there any side effects/trade-offs associated with any of these measures? (Probe: increased biodiversity, increased deer population/numbers, changes in any other wildlife species, long-term maintenance costs)
5. Are you aware of any agri-environment schemes (e.g. woodland creation/tree planting schemes/re-wilding) in the local area?
6. Do you think these schemes might affect deer/ ticks? (Probe: increase or decrease in deer numbers, increase/decrease ticks)
7. Are there any ways in which your estate or farm is currently or going to receive environmental grants for enhance biodiversity? If so, do you think these will affect ticks?

**Other questions and future contact**

1. Is there anything we have not covered that you want to add?
2. Are there other ways you might want to contribute to the project? (Probe: tick reporting, seasonality tick-bite survey, fine-scale mapping exercise etc.)
3. Can you think of anyone else that could/would like to contribute to the project?

Many thanks for your participation in our research
