# Supplementary material for: Exploring stakeholder perceptions and priorities related to reducing tick-related public health risks in natural environments of the United Kingdom
Source: BMC Public Health. 2025 Oct 2;25:3300. doi: 10.1186/s12889-025-24500-7 (PMC12492900; doi:10.1186/s12889-025-24500-7)
Supplement: Supplementary file 4 — Supplementary Material 4 [file 12889_2025_24500_MOESM4_ESM.docx]

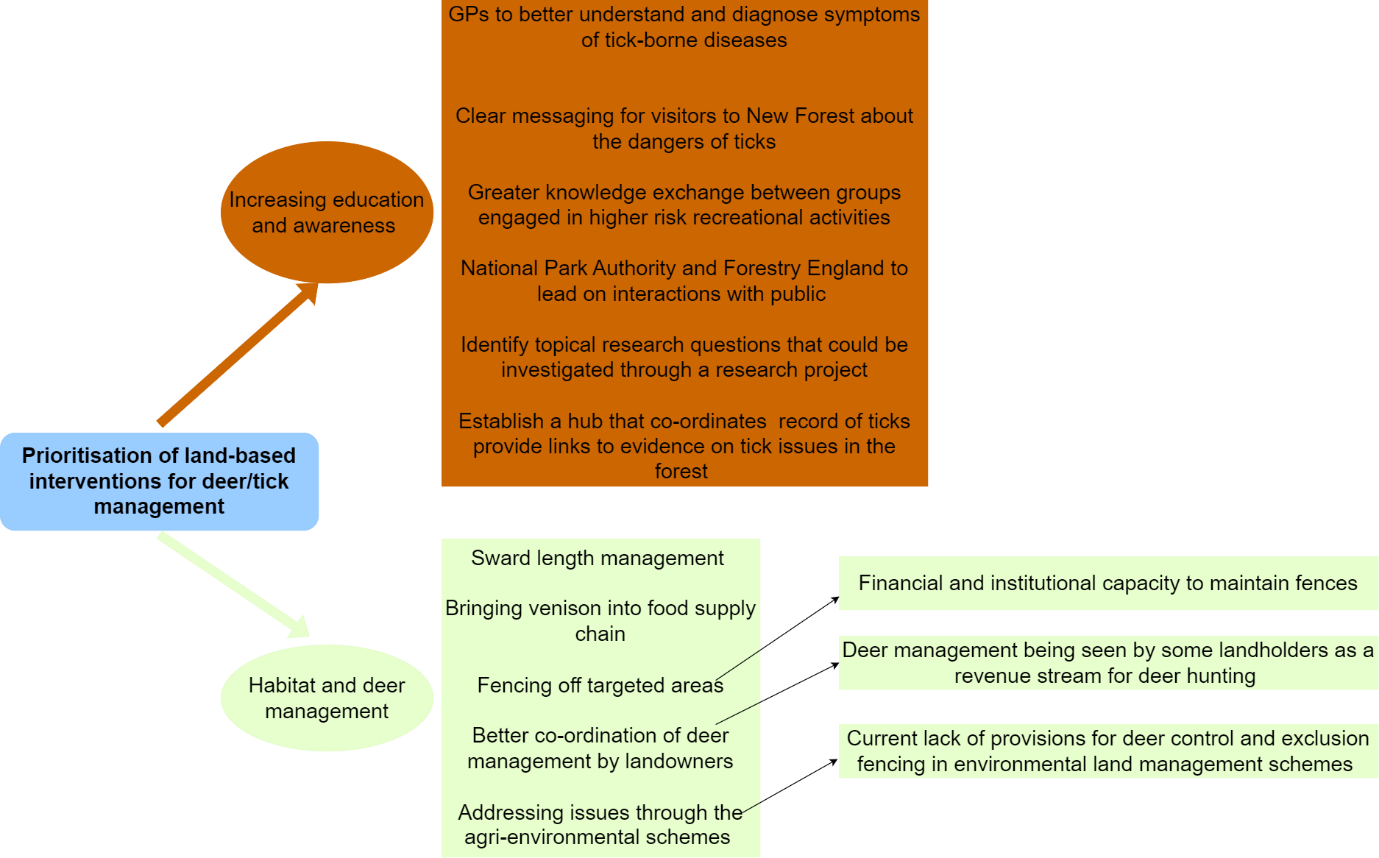


Fig.5. Summary perspectives on options for woodland and wildlife management

**
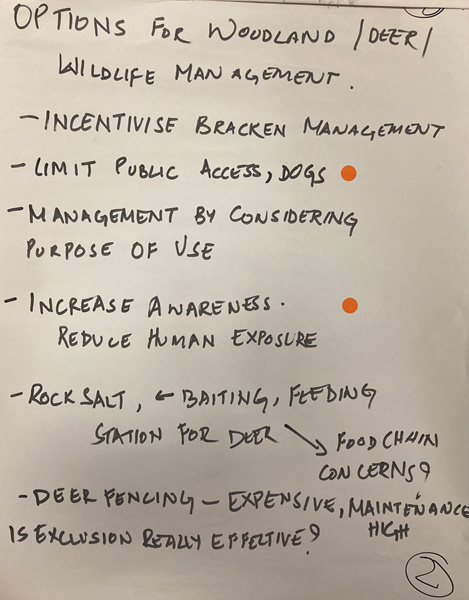

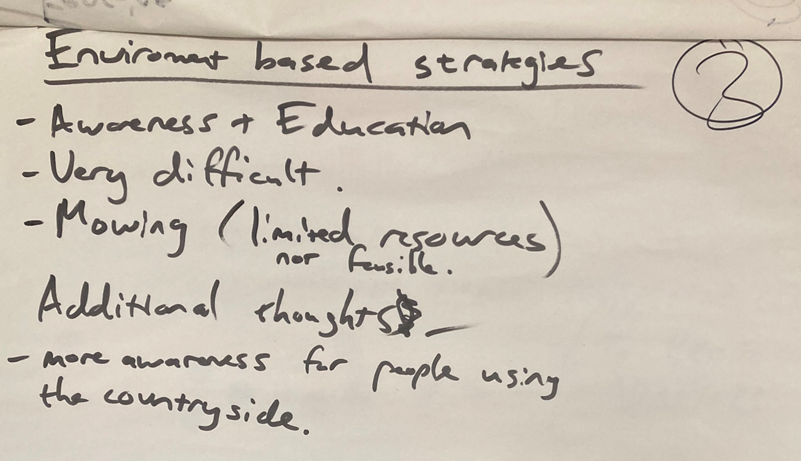
**

Fig.6. An example worksheet of potential environment-based interventions (New Forest workshop)
